# Different dose series of human papillomavirus vaccine in young females: a pair-wise meta-analysis and network meta-analysis from randomized controlled trials

**DOI:** 10.3389/fpubh.2023.1152057

**Published:** 2023-09-21

**Authors:** Li Kemin, Zhang Mengpei, Zeng Jing, Yin Rutie

**Affiliations:** ^1^The Department of Obstetrics and Gynecology, West China Second University Hospital of Sichuan University, Chengdu, China; ^2^Key Laboratory of Birth Defects and Related Diseases of Women and Children (Sichuan University), Ministry of Education, Chengdu, China

**Keywords:** HPV vaccine, different dosage series, randomized controlled study, meta-analysis, network

## Abstract

**Objective:**

To investigate the application value of different dose of HPV vaccine in young females.

**Data sources:**

The following databases were searched: Cochrane Library, PubMed, Embase, Web of Science, SINOMED, and Wanfang Data, from the establishment of the database to August 1st, 2022.

**Study eligibility criteria:**

The inclusion criterias were: healthy young women younger than 25 years old as the research object, randomized controlled study as the research type, and the efficacy and safety of single-dose, two-dose or three-dose HPV vaccines as the intervention measures and research endpoints.

**Study appraisal and synthesis methods:**

Meta-analysis was performed to analyze the protective effects of single-dose, 2-dose and 3-dose HPV vaccine series on young females.

**Results:**

A total of eight eligible studies involving 16 publications were included. There is no difference in the immunogenicity between the 2-dose and 3-dose series within 12 months after the last dose of HPV vaccine. However, 3-dose series was better than the 2-dose series, which performed better than the single-dose vaccine, after 12 months. With respect to the prevention of HPV16/18 infection or HPV31/33/45 infection, the single-dose vaccine worked better than 2-dose or 3-dose series.

**Conclusions:**

The present study showed that the immunogenicity of low-dose HPV vaccine was significantly less, but it reduced the risk of high-risk HPV infection. The low-dose HPV vaccine series may not offer a preventive effect on cervical lesions, though it needs to be further confirmed by additional studies.

## Highlights

- Different HPV vaccine dose for young females.
- Low-dose HPV vaccine may be perfect.
- The results is beneficial to promote the application of HPV vaccine.

## 1. Introduction

The Human Papillomavirus and Related Diseases Report, China, 2021 released by the Catalan Institute of Oncology (ICO) showed that there were nearly 110,000 new cases of cervical cancers each year, with nearly 60,000 deaths in China. The infection rate of the top 10 high-risk human papillomaviruses (HR-HPV) was as high as 97.2%, and a persistent infection of HR-HPV was the culprit behind the cervical cancer (1, 2). HPV vaccines have been widely used clinically. One hundred and ten countries and regions around the world have incorporated HPV vaccines into their national immunization programs until Oct 2020. Global HPV vaccine coverage is increasing year by year. In some countries, HPV vaccine coverage has reached 90% (2).

In 2018, the World Health Organization (WHO) called for the elimination of cervical cancer worldwide, requiring no <90% HPV vaccination rate of young girls under the age of 15. The widespread adoption of HPV vaccine has dramatically reduced the incidence of cervical lesions. A systematic review published by the Lancet in 2019 showed that after 5–9 years of HPV vaccine (bivalent and quadrivalent) in a total of 60 million people in 14 countries around the world, the incidence of cervical intraepithelial neoplasia (CIN) 2+ was significantly reduced by 51 and 31% in women aged from 15 to 19 and 20 to 24 years, respectively (3). However, at present, the insufficient supply of HPV vaccine and the economic burden of people in low-income countries that do not include HPV vaccine in the national immunization plan make the dose and effectiveness of HPV vaccine a new research hotspot. Studies by Bruni et al. (2) have shown that the single-dose HPV vaccine was found to be more feasible in the low- and middle-income countries and may achieve high coverage faster. A Cochrane systematic review has studied the efficacy and safety of different HPV vaccine formulations in various gender and age groups, and the results suggested a no significant difference in the vaccine efficacy between the 2-dose and 3-dose series in young girls (4). A systematic review of non-randomized controlled studies revealed no significant immunological difference between the 2-dose and 3-dose HPV vaccine series in high-income countries (5). Another systematic review of randomized controlled studies investigated the safety and efficacy of the single-dose HPV vaccine and found that it was as effective as that of the multiple doses in preventing HPV infection in healthy young females (6).

At present, there is a lack of high-quality systematic reviews on the effectiveness of the single-dose, 2-dose, and 3-dose HPV vaccines. In this pursuit, this study retrieved the data related to the published randomized controlled studies of HPV vaccines using various doses, and for the first time, systematically studied the preventive effect of the three HPV vaccine dose on cervical lesions in young females in terms clinical efficacy. Our study could serve as a data reference for promoting the broader adoption of HPV vaccine, and further improve its coverage.

## 2. Materials and methods

A pair-wise meta-analysis and network meta-analysis were performed following the Preferred Reporting Items for Systematic Reviews and Meta-Analyses (PRISMA) guidelines (7, 8).

### 2.1. Literature search

**Figure 1:**
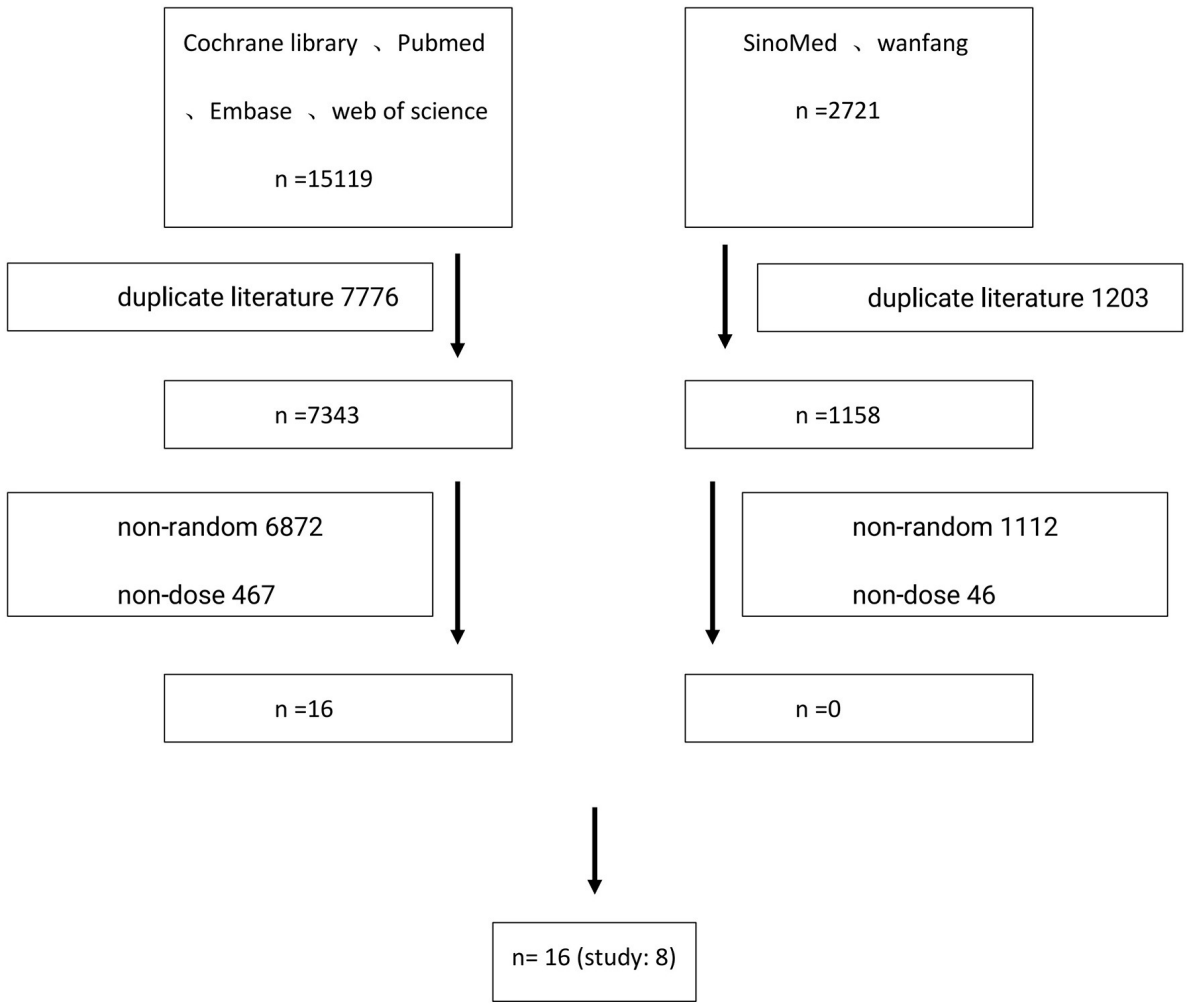
Flowchart of literature search.

According to the search strategy using Medical Subject Headings (MeSH) terms, the following databases were searched: Cochrane Library, PubMed, Embase, Web of Science, SINOMED, and Wanfang Data, from the establishment of the database to August 1st, 2022, by two researchers independently. Any disagreement would be discussed and resolved between them. If not, a third methodology researcher was consulted to reach a final agreement. Search keywords included: HPV, human papillomavirus, vaccine, immunogenicity, randomized controlled studies, and cervical lesions. The detailed inspection strategy is listed in Table 1, and the literature screening process is shown in Figure 1.

**Table 1:** Literature search strategy [for example: Medline (ovid)].

#1 Randomized controlled trial	#11 Papillomavirus infections
#2 Controlled clinical trial	#12 Papilloma viridae
#3 Randomized	#13 #8 or #9 #10 or #11 or #12
#4 Randomly	#14 Immunization
#5 Trial	#15 Vaccin^*^ or immuni^*^
#6 Groups	#16 Vaccine
#7 #1 or #2 or #3 or #4 or #5 or #6	#17 Vaccination
#8 HPV	#18 #14 or #15 or #16 or #17
#9 Human papillomavirus	#19 #7 and #14 and #18
#10 Papillom^*^	

* Unlimited truncation search.

### 2.2. Inclusion and exclusion criteria

The inclusion and exclusion criteria were set according to PICOS (patients, intervention, comparison, outcomes, and study design) principle. The inclusion criterias were: healthy young women younger than 25 years old as the research object, randomized controlled study as the research type, and the efficacy and safety of single-dose, two-dose, or three-dose HPV vaccines as the intervention measures and research endpoints. Efficacy included the immunogenicity and clinical efficacy in preventing HPV infection. Immunogenicity was defined as the geometric mean titers (GMTs) of HPV neutralizing antibodies. HPV vaccines were bivalent, quadrivalent, or non-avalent. The included studies were published studies in English or Chinese.

The study objects older than 25 years old or those in males were not included in this study. The subgroup analysis of randomized controlled trials comparing the efficacy and safety of HPV vaccines using HPV vaccines vs. placebo were not included in this study.

### 2.3. Data extraction

The two scholars extracted the data according to the designed data extraction table and then cross-checked the results. Disagreements were resolved through discussion or with the help of peer experts, if necessary. The data information included the basic characteristics such as study authors, time, study center, study type, intervention measures, study subjects, sample size, and age. It included efficacy and safety indicators such as the short-term immunogenicity within 12 months after the last dose of HPV vaccination, the long-term immunogenicity of over 12 months, clinical indicators of HPV infection prevention (infection rate of various HPV subtypes), toxicity and side effects. It also included the methodological indicators such as randomization plan, blinding plan, and research reports.

### 2.4. Risk of bias and quality assessment of included studies

The risk of bias and quality of included studies were assessed using the randomized controlled study quality assessment tool recommended by the Cochrane Collaboration (9). The evaluation indicators included the following seven items: (1) Random sequence generation (selection bias); (2) Allocation concealment (selection bias); (3) Blinding of participants and personnel (performance bias); (4) Blinding of outcome assessment (detection bias); (5) Incomplete outcome data (attrition bias); (6) Selective reporting (reporting bias); and (7) Other bias. The risk of bias and quality assessment was conducted item-by-item for each study that met the inclusion criteria.

### 2.5. Research endpoints

The research endpoints were immunogenicity and clinical efficacy in preventing the HPV infection. In terms of the immunogenicity of the HPV vaccine, the studies were divided into a short-term group within 12 months and a long-term group of over 12 months after the last dose of HPV vaccination. According to HPV genotypes, the studies were divided into HPV 16 and HPV 18. In terms of the clinical effect of preventing HPV infection, the patients were followed up for more than 1 year after HPV vaccination, and they were divided into HPV16/18 and HPV31/33/45 according to HPV subtypes.

### 2.6. Data analysis

The mean and standard deviation (M + SD) values of the geometric mean titers of HPV neutralizing antibodies were extracted in this study. If the reported results were mean and 95% confidence interval (M + 95% CI), the RevMan 5.4 tool was used to convert the results to M + SD format.

The ADDIS 1.16.8 software (Aggregate Data Drug Information System) was employed for meta-analysis. The odds ratio was used for enumeration data, while the mean difference was adopted for measurement data. First, the chi-square test was used to test the heterogeneity, and *I*^2^ was used to evaluate the heterogeneity. An *I*^2^ ≤ 50% was considered as small heterogeneity and the meta-analysis was considered to be feasible. *I*^2^ > 50% was considered as substantial heterogeneity. The cause of heterogeneity was analyzed, and meta typing was performed after excluding the clinical heterogeneity. When only two intervention measures were combined, a pair-wise meta-analysis (DerSimonian-Laird random effect meta-analysis) was used, and a Markov Chain Monte Carlo Network meta-analysis was conducted when three intervention measures were combined.

## 3. Research results

### 3.1. Basic characteristics of included studies

A total of 17,840 primary screening documents (15,119 in English and 2,721 in Chinese) were retrieved using the search strategy with MeSH terms. After removing the duplicate documents, non-randomized controlled studies and other documents that failed the inclusion criteria, a total of eight studies were included, involving 16 publications (10–25). A total of 32,423 young females aged in the range of 9–25 were included. Six studies reported the short-term immunogenicity of HPV16/18 subtypes (11–16), eight studies reported the long-term immunogenicity of HPV16/18 subtypes (10–17), while two studies reported the clinical efficacy of the HPV vaccine in preventing the HPV infection (10, 17). The basic characteristics of the included studies are shown in Table 2.

**Table 2:** Basic characteristics of included studies.

**References**	**Dose**	**Years**	**Sample**	**HPV vaccine**	**Primary outcomes**
Romanowski et al. (16)	2-dose vs. 3-dose	9–25	960	Bivalent	Immunogenicity, safety
Krajden et al. (13)	2-dose vs. 3-dose	9–26	824	Bivalent	Immunogenicity, safety
Dobson et al. (11)	2-dose vs. 3-dose	9–13	520	Quadrivalent	Immunogenicity, safety
Leung et al. (14)	2-dose vs. 3-dose	9–14	1,075	Bivalent/quadrivalent	Immunogenicity, safety
Lversen et al. (15)	2-dose vs. 3-dose	9–14	1,518	9-Valent	Immunogenicity, safety
Huang et al. (12)	2-dose vs. 3-dose	9–14	1,447	Bivalent	Immunogenicity, safety
Sankaranarayanan et al. (17)	1-dose vs. 2-dose vs. 3-dose	10–18	17,729	Quadrivalent	Incidence
Bhatla et al. (10)	2-dose vs. 3-dose	10–14	9,327	Quadrivalent	Immunogenicity, safety, incidence

### 3.2. Risk of bias and quality assessment of included studies

All the eight studies were randomized controlled studies, seven studies clearly stated the randomization design (10–12, 14–17), and three studies were blinded (11, 14, 16). All the eight studies were high-quality randomized controlled studies. The results of risk of bias and quality assessment of the included studies are shown in Table 3.

**Table 3:** Bias and quality assessment.

	**Romanowski et al. (16)**	**Krajden et al. (13)**	**Dobson et al. (11)**	**Leung et al. (14)**	**Lversen et al. (15)**	**Huang et al. (12)**	**Sankaranarayanan et al. (17)**	**Bhatla et al. (10)**
1. Random sequence generation (selection bias)	Y	N	Y	Y	Y	Y	Y	Y
2. Allocation concealment (selection bias)	Y	N	N	Y	N	N	N	N
3. Blinding of participants and personnel (performance bias)	Y	N	Y	Y	N	N	N	N
4. Blinding of outcome assessment (detection bias)	Y	N	Y	Y	N	N	N	N
5. Incomplete outcome data (attrition bias)	Y	Y	Y	Y	Y	Y	Y	Y
6. Selective reporting (reporting bias)	Y	Y	Y	Y	Y	Y	Y	Y
7. Other bias	Y	?	?	Y	Y	?	?	?

Note: ?: The included research literature does not clearly indicate whether there are other biases.

### 3.3. Short-term immunogenicity of different HPV vaccines

**Figure 2:**
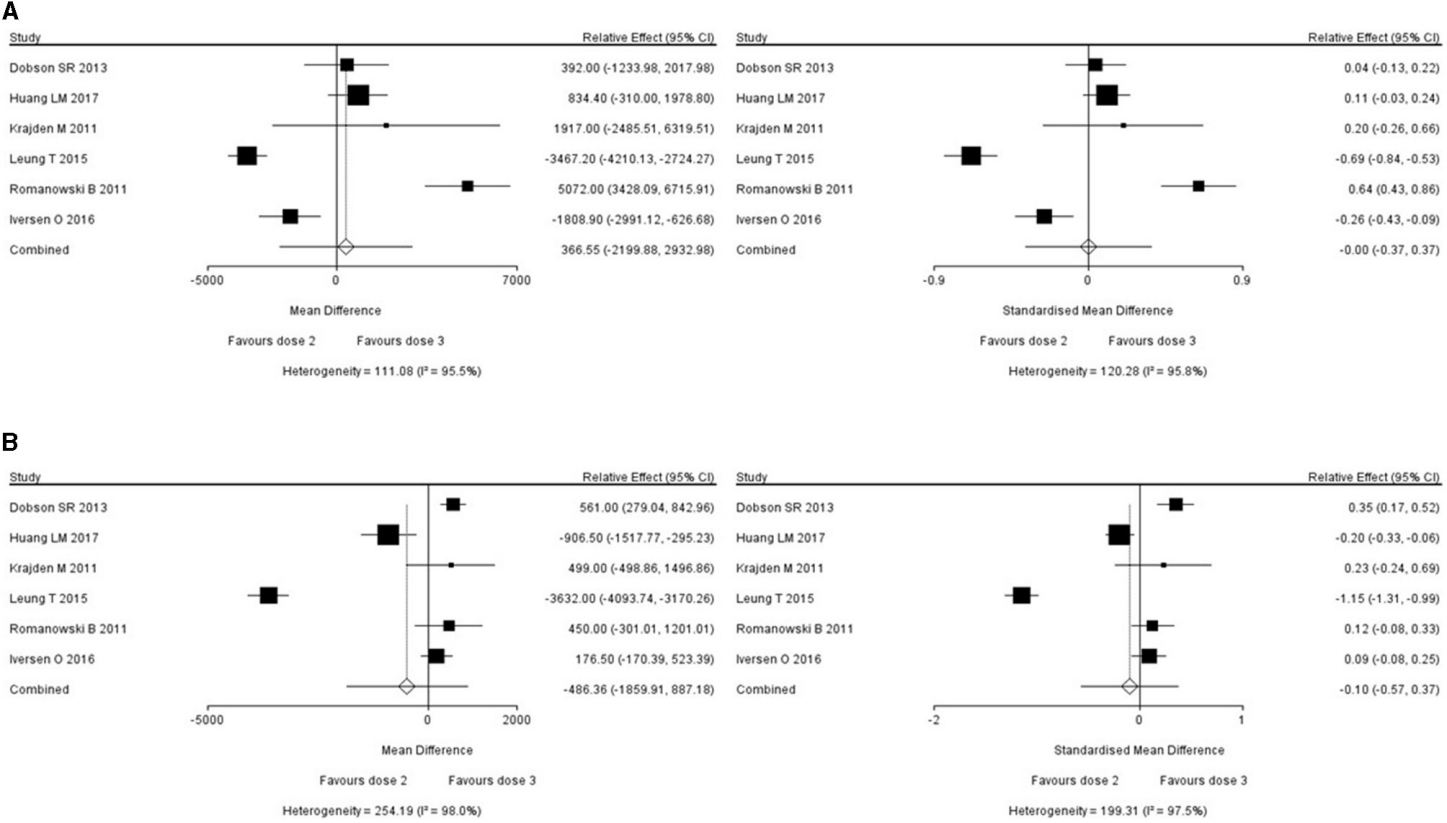
Immunogenicity within 12 months of vaccination (**A**: HPV 16, **B**: HPV 18).

Six studies reported the short-term immunogenicity of HPV16/18 subtypes (11–16), all of which were studies comparing the efficacy and safety of 2- and 3-dose series. For studies with an *I*^2^ value of above 50% (which meant large heterogeneity), the reasons for the heterogeneity were investigated to exclude the clinical heterogeneity, and then the meta typing was performed. When combining two interventions, a pair-wise meta-analysis and DerSimonian-Laird Random effect meta-analysis was adopted. The relative effect and 95% CI of short-term immunogenicity of HPV16 and HPV18 were found to be 366.55 (−2199.88, 293.98) and −486.36 (−1859.91, 887.18). No statistically significant difference between the 2- and 3-dose series was observed in terms of short-term immunogenicity. The short-term immunogenicity of the two HPV vaccine formulations against HPV16 and HPV18 is shown in Figure 2.

### 3.4. Long-term immunogenicity of different HPV vaccines

Eight studies reported the long-term immunogenicity of HPV16/18 subtypes (10–17), seven of which compared the efficacy and safety of 2-dose with 3-dose series (10–16) and one study compared the efficacy and safety of single-, 2- and 3-dose HPV vaccines (17). When combining the three intervention measures, a Markov Chain Monte Carlo Network meta-analysis was adopted.

Consistency is good. Consistency model for HPV 16 shows the OR value and 95%CI of dose 1 vs. dose 2 is −4173.7(−10983.06, 19185.03), the OR value and 95%CI of dose 1 vs. dose 3 is −5333.4(−12425.87, 420.02), the OR value and 95%CI of dose 2 vs. dose 3 is −1159.37(−4060.16, 1344.65). Consistency model for HPV 18 shows the OR value and 95%CI of dose 1 vs. dose 2 is −778.46(−2631.63, 925.83), the OR value and 95%CI of dose 1 vs. dose 3 is −1040.01(−2986.96, 648.22), the OR value and 95%CI of dose 2 vs. dose 3 is −263.18(−1069.69, 398.60). Inconsistency model results show that the stability of the research results is good. Inconsistency model for HPV 16 shows the OR value and 95%CI of dose 1 vs. dose 2 is −422.68(−11051.02, 17542.04), the OR value and 95%CI of dose 1 vs. dose 3 is −5391.51(−12651.41, 501.02), the OR value and 95%CI of dose 2 vs. dose 3 is −1177.56(−4278.95, 1463.41). Inconsistency model for HPV 18 shows the OR value and 95%CI of dose 1 vs. dose 2 is −793.15(−12602.02, 928.05), the OR value and 95%CI of dose 1 vs. dose 3 is −1054.96(−2976.77, 600.55), the OR value and 95%CI of dose 2 vs. dose 3 is −259.44(−1042.52, 394.56).

**Figure 3:**
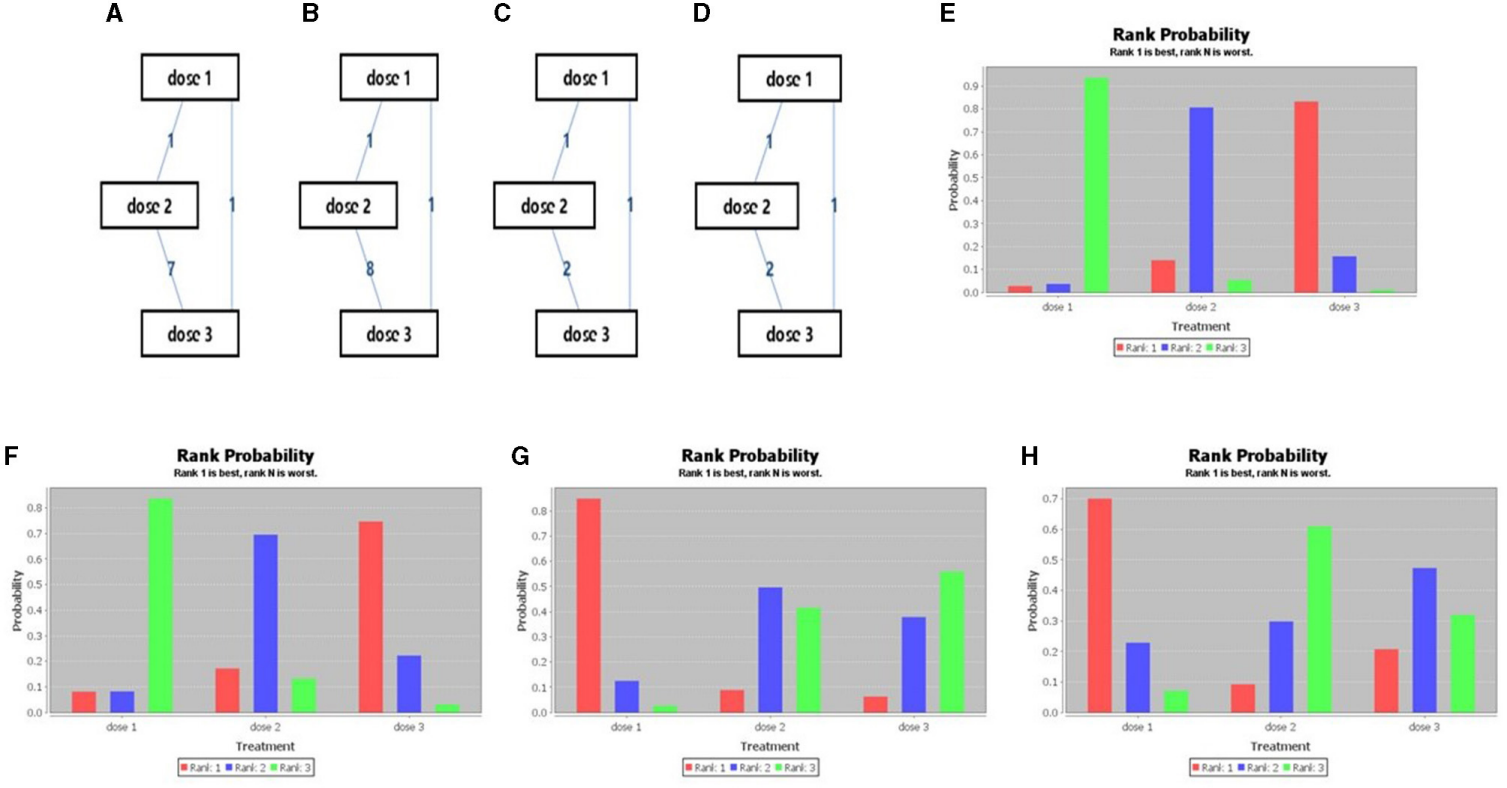
Network meta-analysis evidence network diagram (**A**: HPV 16, **B**: HPV 18, **C**: incidence of HPV 16/18, **D**: incidence of HPV 31/33/45) and Sequence of effects of HPV dosage forms (**E**: HPV 16, **F**: HPV 18, **G**: incidence of HPV 16/18, **H**: incidence of HPV 31/33/45).

**Figure 4:**
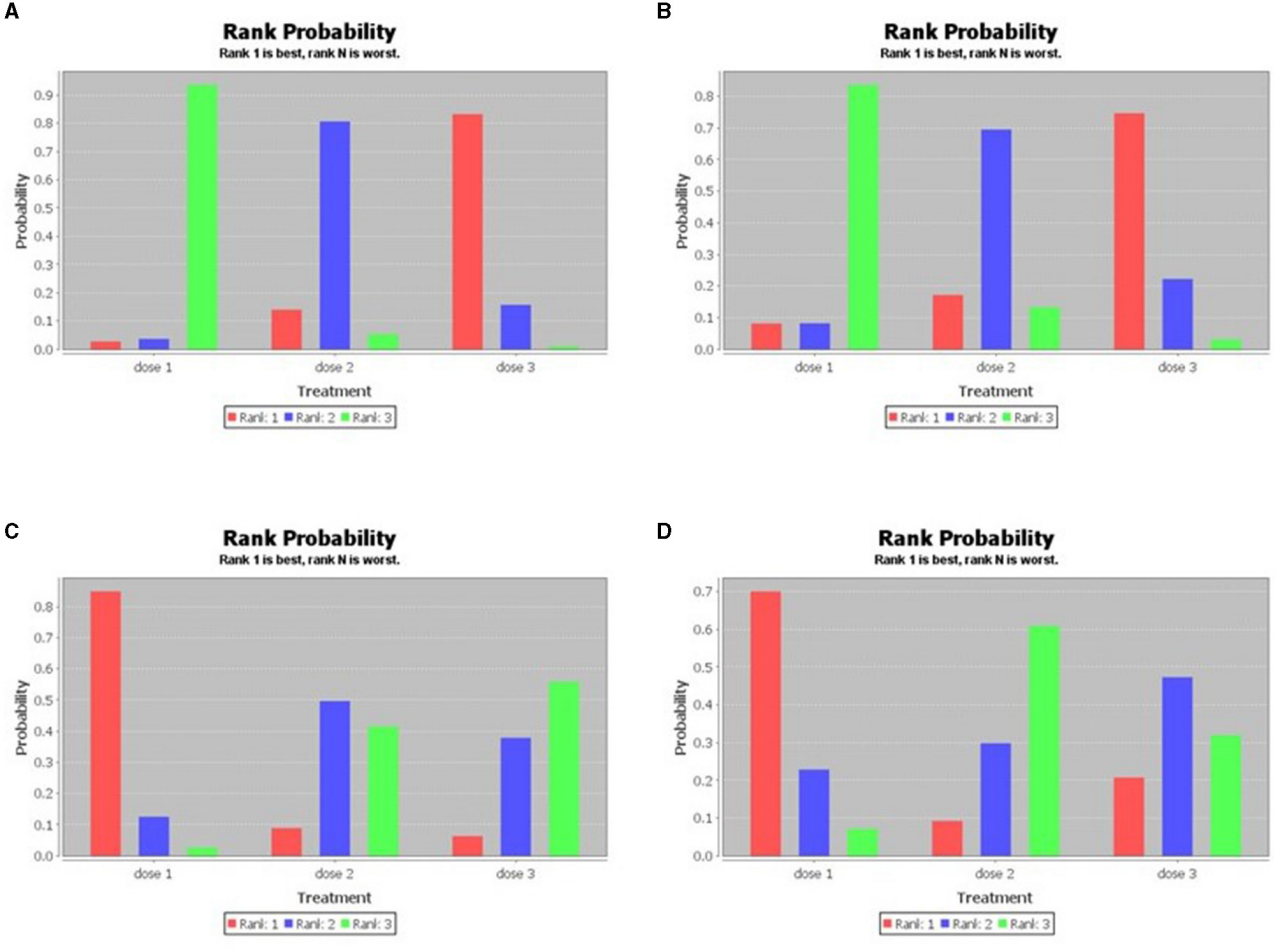
Sequence of effects of HPV dosage forms (the consistency test of the network meta-analysis; **A**: HPV 16, **B**: HPV 18, **C**: incidence of HPV 16/18, **D**: incidence of HPV 31/33/45).

Beyond 12 months of the last dose of vaccination, it was observed that the 3-dose series was better than that of 2-dose series, and 2-dose series was better than single-dose vaccine considering the immunogenicity against HPV16 and HPV18. The evidence map and ranking map of the network meta-analysis is shown in Figures 3, 4.

### 3.5. Clinical effect of HPV vaccine in preventing HPV infection

Two studies reported the HPV infection rates after a minimum follow-up of 3 years after HPV vaccination. One study compared the effects of 2- and 3-dose series, and another study compared the effects of single-, 2-, and 3-dose series.

Consistency is good. Consistency model for HPV 16/18 shows the OR value and 95%CI of dose 1 vs. dose 2 is 1.75(0.72, 4.34), the OR value and 95%CI of dose 1 vs. dose 3 is 1.90(0.76, 4.88), the OR value and 95%CI of dose 2 vs. dose 3 is 1.08(0.41, 2.79). Consistency model for HPV31/33/45 shows the OR value and 95%CI of dose 1 vs. dose 2 is 1.25(0.82, 1.88), the OR value and 95%CI of dose 1 vs. dose 3 is 1.14(0.77, 1.74), the OR value and 95%CI of dose 2 vs. dose 3 is 0.92(0.62, 1.39). Inconsistency model results show that the stability of the research results is good. Inconsistency model for HPV 16/18 shows the OR value and 95%CI of dose 1 vs. dose 2 is 1.75(0.67, 4.92), the OR value and 95%CI of dose 1 vs. dose 3 is 2.03(0.83, 5.27), the OR value and 95%CI of dose 2 vs. dose 3 is 1.14(0.42, 3.14). Inconsistency model for HPV31/33/45 shows the OR value and 95%CI of dose 1 vs. dose 2 is 1.24(0.83, 1.87), the OR value and 95%CI of dose 1 vs. dose 3 is 1.15(0.78, 1.77), the OR value and 95%CI of dose 2 vs. dose 3 is 0.93(0.62, 1.40).

In the prevention of HPV16/18 infection, single-dose vaccine was superior to 2- and 3- dose series, and the 2-dose series was superior to 3-dose series. While in the prevention of HPV31/33/45 infection, the single-dose vaccine was superior to the 2- and 3-dose series, and the 3-dose series was superior to the 2-dose series. The evidence and ranking maps of the network meta-analysis is shown in Figures 3, 4.

## 4. Discussion

### 4.1. Main findings

The immunogenicity of low-dose HPV vaccine was significantly less, but it reduced the risk of high-risk HPV infection. The low-dose HPV vaccine series may not offer a preventive effect on cervical lesions, though it needs to be further confirmed by additional studies.

### 4.2. Strengths and limitations

The present study included randomized controlled studies involving young healthy females, large sized samples, and mostly international multi-center studies, in order to reduce the correlation bias due to confounding factors such as ethnicity, sample size, and age. The results of our study support the fact that the reduced doses of HPV vaccine may not affect the ability of the HPV in preventing the cervical lesions, and it is beneficial to promote the application of HPV vaccine, expanding the coverage of HPV vaccination, and achieving the goal of eliminating cervical cancer called by the WHO. However, the present study still presents some limitations. First, the heterogeneity between the studies was large. The follow-up time nodes of immunogenicity indicators and clinical indicators of HPV infection were different, and the sample sizes were different, thereby increasing the heterogeneity of the studies. Only one study compared three HPV vaccine formulations. The time points of HPV vaccination were different in different studies, increasing the unreliability of the study results. HPV Persistent Infection and CIN Incidence are Closely Related to Cervical Lesions. However, only two studies reported the HPV infection rates after a minimum follow-up of 3 years after HPV vaccination, there were no studies with persistent HPV infection or incidence of CIN as the end point. The present study did not investigate the effect of different doses of HPV vaccine on the incidence of cervical lesions, and to date, no related randomized controlled trials (RCT) studies have been reported. These factors may significantly affect the accuracy of the research conclusions. 2#, 4#, or 9# vaccines have different immune targets and use immune adjuvants, it may be not suitable for analyzed these three different vaccines combinedly. We attempted to perform a subgroup analysis by HPV vaccine type, because of data limitations, we finally had no choice but to give up. We hope that more research appears later, we will update this part of the problem.

### 4.3. Comparison with existing literature

Human papilloma virus (HPV) vaccination is an effective primary preventive measure against HPV infection and infection-related diseases (26). HPV vaccine induces a humoral immune response, and the produced neutralizing antibodies can bind to the virus antigen when HPV enters the body, thereby preventing the HPV infection. The occurrence and progression of cervical pre-cancer may be blocked by preventing the primary HPV infection and reducing the persistent HPV infection. The antibodies induced by the vaccine can penetrate the blood vessel wall and accumulate in the local epithelial tissue in a high concentration. When the HPV particles reaches the basal layer cells via a micro-wound in the mucosal epithelium, antibodies located in the epithelial tissue can bind to the viral particles and neutralize them. Studies at home and abroad have shown that after the completion of the whole course of immunization with bivalent, quadrivalent or nonavalent HPV vaccines, high rates of vaccine-related antibody seroconversion and serological antibody titers (96–100%) were observed (27–29). The results of the clinical studies in China showed that females aged 9–17 exhibited stronger immune responses after being vaccinated with bivalent and quadrivalent HPV vaccines, and their serological antibody titers were 1.42–3.00 times higher than those of females aged 18–26, whose antibody titers were similar to women aged 26–45 (30–32). A phase III clinical trial exhibited that the antibody titers of HPV (6/11/16/18) in females aged 16–17 after receiving the non-avalent HPV vaccine were higher than those aged 18–26, while their immune responses were comparable to those receiving the quadrivalent HPV vaccine (33).

Some studies have shown that by reducing the number of HPV vaccine doses may be beneficial for expanding the HPV vaccination coverage (2). Meta-analysis demonstrated that the immunogenicity of HPV vaccines of various dosage forms could be similar (5, 6). However, these studies were meta-analyses of non-randomized controlled studies or systematic reviews comparing only two dosage forms. There is currently a lack of quality and evidence-based investigation for the superiority of the three HPV vaccine formulations. The present study is the first to discuss the effectiveness of the single-dose, 2-dose, and 3-dose HPV vaccines in young females in a network meta-analysis format. We found no statistically significant difference between the 2- and 3-dose series was observed in terms of short-term immunogenicity, that is, the short-term immunogenicity of 2-dose is similar with 3-dose's short-term immunogenicity. Consistency is good, and inconsistency model results show that the stability of the research results is good in long-term immunogenicity of different HPV vaccines. Beyond 12 months after the last dose, the 3-dose series displayed the strongest immunogenicity, followed by the 2-dose series and the single-dose one, respectively.

HPV vaccines have exhibited 87.3–100.0% protective efficacy in the prevention of HPV type-related diseases in clinical trials. The quadrivalent HPV vaccine has a good protective effect in females aged 18–25. One study with 78-month follow-up for Chinese females aged 20–45 showed that the quadrivalent HPV vaccine provided 100% protection against HPV16/18-related CIN 2/3, adenocarcinoma *in situ* (AIS), and cervical cancer (29). The non-avalent HPV vaccine showed a comparable protective efficacy as that of the quadrivalent HPV vaccine against the HPV6/11/16/18-related persistent infection and cervical cancer in females aged 16–26 (32). The non-avalent HPV vaccine exhibited 100% protective efficacy against HPV 31/33/45/52/58-related CIN1+ in the subgroup of East Asian females aged 16–26, and showed 95.8% protective efficacy against the 6-month and above persistent HPV 31/33/45/52/58-related infections in cervix, vagina, vulva, and anus (34). However, the differences in the preventive effect of various HPV vaccine dose on HPV infection remains controversial. Few studies compared the prevention of HPV infection between the different dose of HPV vaccines. Consistency is good, and inconsistency model results show that the stability of the research results is good in clinical effect of HPV vaccine in preventing HPV infection. The present meta-analysis showed that the single-dose vaccine was the most effective in preventing HPV16/18 infection, followed by the 2-dose and the 3-dose series, respectively. In terms of preventing the HPV31/33/45 infection, the single-dose performed the best, followed by the 3-dose series, and the 2-dose series, respectively. The results of preventing the HPV infection were inconsistent to the immunogenicity results of HPV vaccine, and presently it is difficult to explain the vaccine mechanism. The reason may be that Antibody responses to a single dose of the HPV vaccine more closely resemble those to a live virus infection, which can persist indefinitely at a relatively steady-state level (35). Other antivirion antibody responses induced by viral infection or live viral vaccines, which present the same type of high density repetitive display of surface epitopes as HPV VLPs, have been shown to persist at essentially constant levels for many decades. Yet the sustained immunological responses observed among single-dose HPV-vaccinated participants were unexpected because it has not been observed with other subunit vaccines. For example, anti-Hepatitis B (HB) positivity in young adults (median age of 25 years) after single-dose HB vaccination was 4% and dropped to 0% after 2 years, with antibody levels below the threshold considered necessary for protection against HBV infection. However, the subviral particles that comprise the HBV vaccine may not be sufficiently “virus-like” to efficiently induce durable antibody responses. It may be also related to factors such as sample size, heterogeneity of studies, age, and ethnicity of the research subjects, and needs further clarification.

## 5. Conclusions and implications

In conclusion, although the low-dose series of the HPV vaccine showed no advantages in terms of immunogenicity, nevertheless they demonstrated clear advantages in preventing the high-risk HPV infection. It is proposed that the low-dose HPV vaccine may not show less protection from HPV. This vaccination strategy can further expand the HPV vaccination rate of young girls under the age of 15. It may have significant value for cervical cancer prevention in developing countries. However, this should be verified by systematically prospective large sample randomized controlled studies on the three formulations, and special attention should be paid to the effect of preventing the cervical lesions.

## Author contributions

Conception: LK and YR. Design and final manuscript approval: All authors. Data collection and manuscript writing: LK and ZM. Data analysis: LK. All authors contributed to the article and approved the submitted version.
